# A machine learning-based radiomics model for prediction of tumor mutation burden in gastric cancer

**DOI:** 10.3389/fgene.2023.1283090

**Published:** 2023-11-06

**Authors:** Tingting Ma, Yuwei Zhang, Mengran Zhao, Lingwei Wang, Hua Wang, Zhaoxiang Ye

**Affiliations:** ^1^ Department of Radiology, Tianjin Cancer Hospital Airport Hospital, Tianjin, China; ^2^ Department of Radiology, Tianjin Medical University Cancer Institute and Hospital, Tianjin, China; ^3^ National Clinical Research Center for Cancer, Tianjin, China; ^4^ Tianjin’s Clinical Research Center for Cancer, Tianjin, China; ^5^ The Key Laboratory of Cancer Prevention and Therapy, Tianjin, China

**Keywords:** radiomics, tumor mutation burden, machine learning, gastric cancer, computed tomograph

## Abstract

**Purpose:** To evaluate the potential of machine learning (ML)-based radiomics approach for predicting tumor mutation burden (TMB) in gastric cancer (GC).

**Methods:** The contrast enhanced CT (CECT) images with corresponding clinical information of 256 GC patients were retrospectively collected. Patients were separated into training set (*n* = 180) and validation set (*n* = 76). A total of 3,390 radiomics features were extracted from three phases images of CECT. The least absolute shrinkage and selection operator (LASSO) model was used for feature screening. Seven machine learning (ML) algorithms were employed to find the optimal classifier. The predictive ability of radiomics model (RM) was evaluated with receiver operating characteristic. The correlation between RM and TMB values was evaluated using Spearman’s correlation coefficient. The explainability of RM was assessed by the Shapley Additive explanations (SHAP) method.

**Results:** Logistic regression algorithm was chosen for model construction. The RM showed good predictive ability of TMB status with AUCs of 0.89 [95% confidence interval (CI): 0.85–0.94] and 0.86 (95% CI: 0.74–0.98) in the training and validation sets. The correlation analysis revealed a good correlation between RM and TMB levels (correlation coefficient: 0.62, *p* < 0.001). The RM also showed favorable and stable predictive accuracy within the cutoff value range 6–16 mut/Mb in both sets.

**Conclusion:** The ML-based RM offered a promising image biomarker for predicting TMB status in GC patients.

## Introduction

Immune checkpoint inhibitors (ICIs), represented by programmed cell death protein 1 (PD-1) and programmed death-ligand 1 (PD-L1) have revolutionized the treatment paradigm and shown exciting efficacy in a variety of solid tumors. Several clinical trials have highlighted the effectiveness and safety of PD-1 inhibitors in the management of gastric cancer (GC) patients (Kang et al., 2017; Fuchs et al., 2018; Taieb et al., 2018). However, the response rate of PD-1 inhibitors is only about 20% for most patients with advanced solid tumors (Chen and Mellman, 2017). Moreover, unlike conventional chemotherapy, some patients treated with ICIs developed multiple novel and complex response patterns such as delayed response, pseudoprogression and hyperprogression (Borcoman et al., 2019). Studies have shown that for GC patients treated with PD-1 inhibitors, the response rate was not significantly related to expression status of PD-1/PD-L1 (De Rosa et al., 2018). Therefore, identifying biomarkers to predict the effectiveness of PD-1 inhibitors remains a significant issue to be addressed for PD-1 inhibitor therapy in GC, which is of great significance for screening patients with potential benefit and reducing side effects.

By utilizing high-throughput sequencing data, The Cancer Genome Atlas (TCGA) has categorized GC into four distinct molecular subtypes: Epstein–Barr virus (EBV), microsatellite instability (MSI), chromosomal instability (CIN), and genomically stable (Cancer Genome Atlas Research, 2014). Subsequently, the role of MSI, EBV infection and tumor mutation burden (TMB) for prediction of treatment efficacy of PD-1 inhibitors have also been proposed by several studies (Topalian et al., 2016; De Rosa et al., 2018; Kim et al., 2018).

TMB is defined as the total count of nonsynonymous mutations per one million bases within tumor tissue. Mutations in driver genes could lead to the development and progression of tumor. In addition, the high amount of gene mutations could facilitate the generation of neoantigen on tumor cells. The neoantigens can be recognized by the autoimmune system and enhance the tumor immunogenicity by activating T cells (Gubin et al., 2015). Therefore, patients with higher TMB tend to derive benefit from immunotherapy. Several studies have shown the potential of TMB for serving as an important predictor for treatment response of ICIs in various types of tumors including GC (Chan et al., 2019). Carbone et al. reported that nivolumab was associated with a higher response rate than chemotherapy in non-small-cell lung cancer (NSCLC) patients with higher TMB, regardless the expression status of PD-L1 (Carbone et al., 2017). The CheckMate 227 study reported that patients of advanced NSCLC with higher TMB had better response to immunotherapy (Reck et al., 2019). In a clinical trial of toripalimab for chemo-refractory GC, patients with higher TMB exhibited better overall survival than those with lower TMB (Wang et al., 2019).

Currently, the assessment and calculation of TMB rely on the next-generation sequencing (NGS) of primary tumor tissue or peripheral blood samples. However, the results of sequencing of circulating tumor DNA are easily influenced by the sample and DNA content. In addition, patients in advanced disease often require multiple biopsies to surveillance changes of TMB. The high heterogeneity of tumor renders the biopsy samples incapable of fully representing the entire landscape of the tumor. Moreover, high cost of NGS is unaffordable for many patients. These reasons limit the widespread application of TMB testing. Therefore, there is a significant necessity to discover a simple and noninvasive approach for assessing TMB.

Currently, computed tomography (CT) servers as the most important tool for diagnosing, preoperative staging, and treatment efficacy assessment for GC. However, the consistency and accuracy of image interpretation varies largely. Moreover, the analysis of CT images predominantly depends on morphological features. However, these features offer only constrained insights into the underlying tumor, failing to fulfill the requirements of the personalized and precision medicine. With the advancement of computing power and graphic processing technologies, artificial intelligence (AI) is being increasingly utilized to analyze large-scale and complex data, including medical imaging such as endoscopic, pathological, and radiological imaging (Bi et al., 2019; Hsiao et al., 2021). Radiomics, which is a branch of AI, refers to a new data process and mining technique that can translate images into high-through quantitative data. The radiomics features have the capability to discern underlying features within tumors, which reflect the characteristics of cellular composition, protein expression, genetic mutations, tumor microenvironment, and heterogeneity. Radiomics has been widely applicated in the fields of tumour segmentation, early screening, tumour staging, prognosis prediction, treatment efficacy evaluation and surveillance (Bi et al., 2019). Moreover, multiple studies have illustrated the value of radiomics in predicting genetic statuses and molecular subtypes.

To date, there is still a dearth of research investigating the value of radiomics methods for predicting TMB in GC. Thus, this study aims to explore the value of radiomics model (RM) for evaluation of TMB in GC.

## Materials and methods

### Patients

From February 2019 to March 2021, 256 consecutive GC patients were enrolled. Based on the timing of the CT scan, the patients were separated into training (n = 180) and validation set (n = 76) at a ratio of 7:3.

The eligibility criteria: 1) patients with a pathologically confirmed diagnosis; 2) CT images were acquired within 2 weeks prior to surgery; 3) TMB testing result available 4) Imaging quality meets the requirements of analysis: a) sufficient distention of gastric cavity; b) No respiratory and peristaltic artifacts were observed in the images. The exclusion criteria: 1) incomplete clinical information; 2) patients underwent treatment prior to CT scan; 3) patients who had other malignant disease.

**FIGURE 1:**
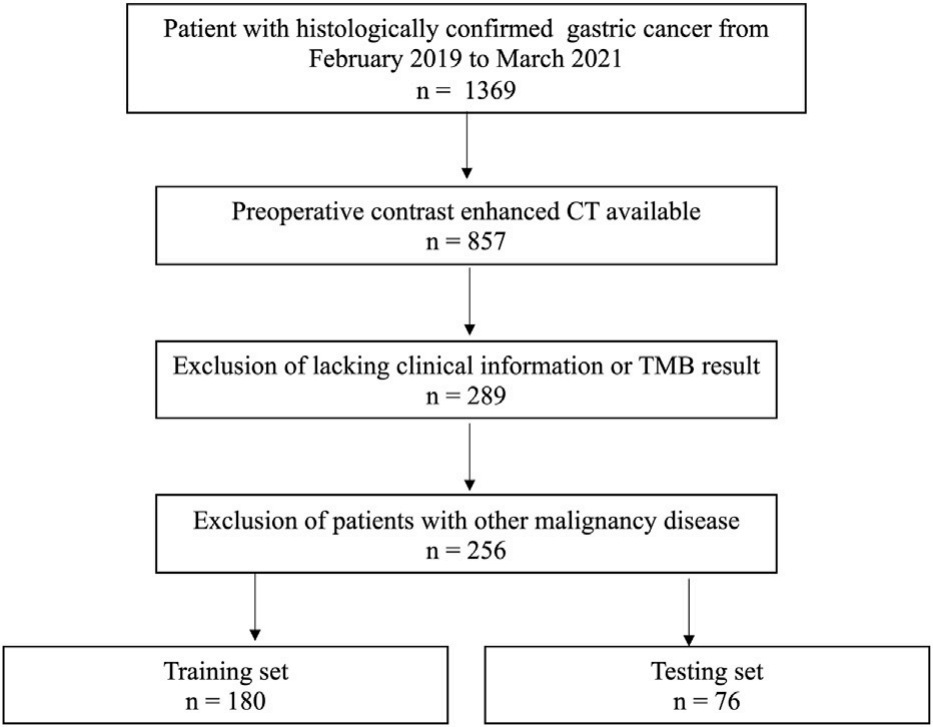
Patients’ enrollment pathway.

Our institutional ethical review board approved the conduction of this study. The need for informed consent was waived. The patient’s enrollment pathway is illustrated in Figure 1.

### TMB testing

A NGS test was performed on genomic DNA isolated from formalin-fixed paraffin-embedded surgically resected tumor samples. A commercial targeted NGS-panel which contained 639 related genes was used and NGS was performed on Illumina Nextseq 500/550 platform (Illumina Inc., San Diego, CA, USA). As recommended by previous studies (Mishima et al., 2019; Lee et al., 2022a), TMB ≥10 mut/Mb was used as cutoff value for defining high-TMB, while TMB <10 mut/Mb was defined as low-TMB.

### Lesion delineation and feature extraction

Abdominal contrast enhanced CT (CECT) images in three phases were used for analyzed (Ma et al., 2017; Gao et al., 2021). The detail of CT image acquisition protocol was shown in the Supplementary Material S1. By using 3D Slicer software (V.5.0.2), the volume of interest (VOI) of each tumor was delineated by 2 radiologists. Feature extraction was carried out using utilizing PyRadiomics 2.2.0 (van Griethuysen JJM et al., 2017). For each phase of original and filtered CECT images, 1,130 features were extracted. The features were categorized into first order statistics, shape, Gray Level Cooccurence Matrix (GLCM), Gray Level Run Length Matrix (GLRLM), Gray Level Size Zone Matrix (GLSZM), Neighbouring Gray Tone Difference Matrix (NGTDM) and Gray Level Dependence Matrix (GLDM). The detail of the radiomics features is described previously (van Griethuysen JJM et al., 2017).

### Radiomics model training

**FIGURE 2:**
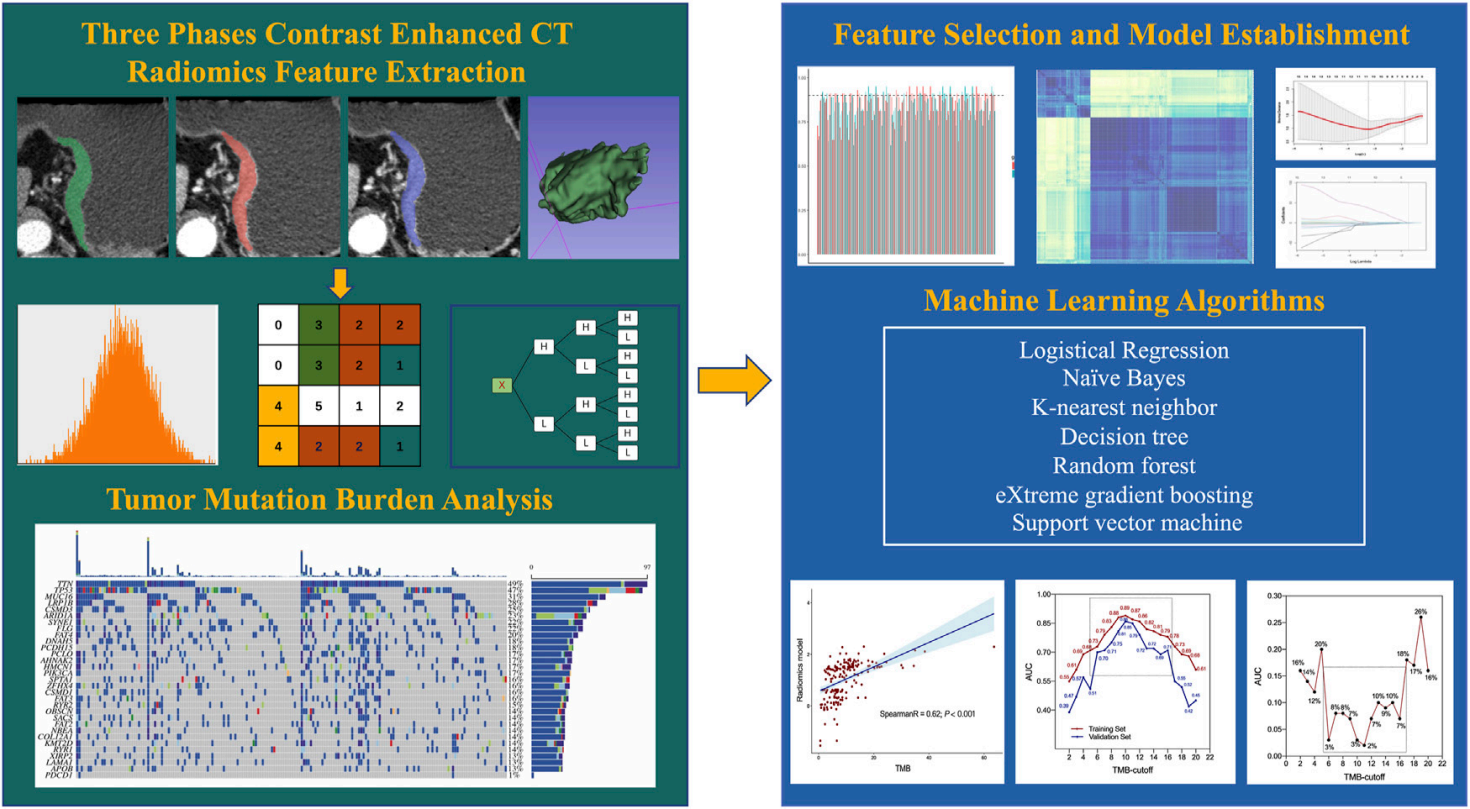
Flowchart of radiomics analysis.

To ensure the robustness and stability, interclass correlation coefficients (ICC) of features were analyzed. Two readers independently performed VOI segmentation on a randomly selected group of 30 patients from the training set. The features were regarded as stable if ICC were higher than 0.9. Next, the correlation between features was analyzed using Spearman’s rank test. We retained one of the two features that showed a correlation coefficient higher than 0.9. Then, features exhibiting a statistically significant between the high-TMB and low-TMB groups were identified by utilizing the Mann-Whitney U test. The least absolute shrinkage and selection operator (LASSO) model was subsequently utilized to find features with best predictive ability. Depending on the regularization weight λ, the LASSO method effectively pulls all regression coefficients toward zero while precisely assigning coefficients of numerous irrelevant features as zero. To determine the ideal λ, we conducted a 10-time cross-validation using a minimization criterion. The optimal λ value was selected based on achieving the lowest cross-validation error. Following the LASSO feature selection, seven additional machine learning (ML) algorithms were employed for RM training: k-nearest neighbor (KNN), decision tree (DT), random forest (RF), support vector machine (SVM), logistic regression (LR), adaptive boosting (AdaBoost) and naïve Bayes (NB). Figure 2 illustrates the overall radiomics procedure.

### Statistical analysis

The predictive power of ML model was determined with the receiver operating characteristic (ROC) curve with corresponding value of the area under the curve (AUC). The correlation between RM and TMB value was evaluated using Spearman’s correlation coefficient. Categorical variables were compared by the Chi-Squared or Fisher exact tests. The Shapley Additive explanations (SHAP) was used to visualize the ML model and quantify the importance of features (Li et al., 2020). All statistical analyses were conducted using R (V.4.2.3).

## Results

### Patients

In total, 256 GC patients including 176 men and 80 women were enrolled. The median age of patients was 60 years old. The overall median value of TMB was 6.5 mut/Mb. The training set contained 60 (33.3%) high-TBM patients, while the validation set contained 19 (25.0%) high-TMB patients. The clinicopathological characteristics of all patients have been summarized in Table 1.

**TABLE 1:** Characteristics of the study population.

Variable	Training set (n = 180)			Testing set (n = 76)		
	High-TMB (n = 60)	Low-TMB (n = 120)	*P*	High-TMB (n = 19)	Low-TMB (n = 57)	*P*
Age			0.55			0.69
< 65	39 (65.0)	83 (69.2)		12 (63.2)	33 (57.9)	
≥ 65	21 (35.0)	37 (30.8)		7 (36.8)	24 (42.1)	
Sex			0.73			0.40
Male	41 (68.3)	85 (70.8)		11 (57.9)	39 (68.4)	
Female	19 (31.7)	35 (29.2)		8 (42.1)	18 (31.6)	
Tumor Site			0.26			0.29
Upper-Middle	23 (38.4)	32 (26.7)		7 (36.9)	16 (28.0)	
Lower	17 (28.3)	37 (30.8)		10 (52.6)	25 (43.9)	
Overlap	20 (33.3)	51 (42.5)		2 (10.5)	16 (28.1)	
Pathologic T stage			0.34			0.74
T_1-2_	13 (21.7)	34 (28.3)		4 (21.1)	10 (17.6)	
T_3-4_	47 (78.3)	86 (71.7)		15 (78.9)	47 (82.4)	
Pathologic N stage			0.92			0.98
N_0_	16 (26.7)	38 (31.7)		6 (31.6)	16 (28.1)	
N_1-3_	44 (73.3)	82 (68.3)		13 (68.4)	41 (71.9)	
Pathologic TMN stage			0.78			0.88
I	9 (15.0)	25 (20.8)		3 (15.8)	7 (12.3)	
II	11 (18.3)	21 (17.5)		6 (31.6)	15 (26.3)	
III	39 (65.0)	71 (59.2)		9 (47.4)	33 (57.9)	
IV	1 (1.7)	3 (2.5)		1 (5.3)	2 (3.5)	
LVI			0.25			0.88
Negative	46 (76.7)	82 (68.3)		14 (73.7)	41 (71.9)	
Positive	14 (23.3)	38 (31.7)		5 (26.3)	16 (28.1)	
PNI			0.49			0.40
Negative	44 (73.3)	82 (68.3)		14 (73.7)	36 (63.2)	
Positive	16 (26.7)	38 (31.7)		5 (26.3)	21 (36.8)	
CEA			0.54			0.31
≥ 5.0 μg/mL	7 (11.7)	18 (15.0)		5 (26.3)	9 (15.8)	
< 5.0 μg/mL	53 (88.3)	102 (85.0)		14 (73.7)	48 (84.2)	
CA19-9			0.59			0.87
≥ 27 U/mL	13 (21.7)	22 (18.3)		4 (21.1)	11 (19.3)	
< 27 U/mL	47 (78.3)	98 (81.7)		15 (78.9)	46 (80.7)	
CA242			0.27			0.54
≥ 20 U/mL	10 (16.7)	13 (10.8)		3 (15.8)	6 (10.5)	
< 20 U/mL	50 (83.3)	107 (89.2)		16 (84.2)	51 (89.5)	
CA72-4			0.49			0.47
≥ 6.9 U/mL	12 (20.0)	19 (15.8)		4 (21.1)	8 (14.0)	
< 6.9 U/mL	48 (80.0)	101 (84.2)		15 (78.9)	49 (86.0)	

In both sets, no significant differences were observed between the high-TBM and low-TMB groups regarding age, sex, tumor site, pathological stage, lymphovascular invasion status, perineural invasion status and the level of four serum biomarkers.

### Radiomics feature screening

**FIGURE 3:**
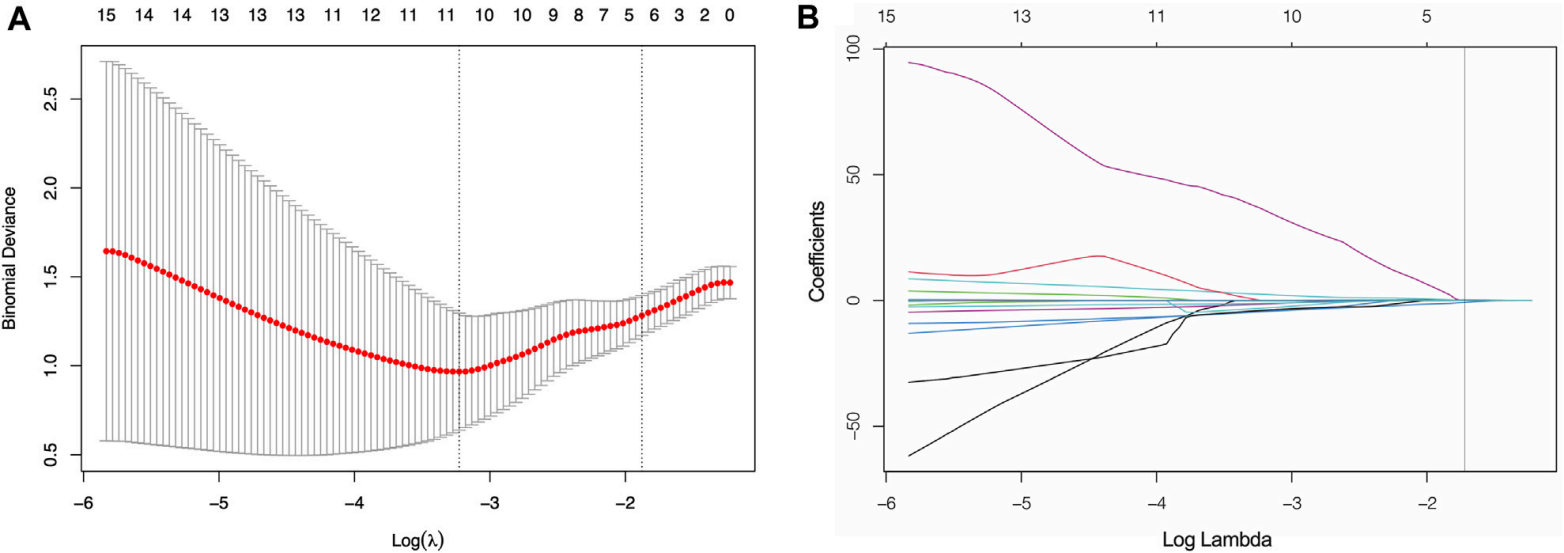
Screening of significant features by LASSO model **(A)** Ten time cross-validation for tuning parameter selection. **(B)** LASSO coefficient profiles.

The VOIs in three phases of CECT images were delineated and a total of 3,390 features were extracted. Firstly, 2,293 features with ICCs <0.90 were excluded. After calculating the correlation coefficients of the remaining 1,097 features, a total of 398 redundancy features were eliminated. Next, 56 features that showed significant differential changes of the remaining 699 features between the high- and low-TMB groups were selected and putted into LASSO model. Finally, twelve features were chosen, including 3 features from AP (log.sigma.1.0 mm first ortder-mean, log. sigma.1.0 mm firstorder-90-Percentile, log. sigma.1.0 mm GLSZM-Dependence Non Uniformity), 6 features from PP (wavelet.HLL-GLSZM-zone entropy, wavelet. LHL-NGTDM-Busyness, wavelet. HLH-firstorder-Kurtosis, wavelet. LHH-GLRLM-Run Entropy, log. sigma.1.0 mm. GLDM-Large Dependence Emphasis, wavelet. HHH-GLCM-joint entropy) and 3 features from DP (maximum 3D diameter, wavelet. HHH-GLRLM-Gray Level NonUniformity Normalized, log. sigma.1.0 mm. GLRLM-High Gray Level Run Emphasis) (Figure 3).

### Predictive performance of radiomics model

To determine the optimal classifier for the establishment of RM, seven other ML algorithms were employed for model training. As shown in Table 2, the AUCs of LASSO, KNN, DT, RF, SVM, LR, AdaBoost and NB were 0.82, 0.70, 0.80, 0.69, 0.79, 0.89, 0.75 and 0.65 respectively. Hence, the LR model [AUC: 0.89, 95% confidence interval (CI): 0.85–0.94] was selected for construction of the RM. The predictive ability of RM was confirmed in the validation set with AUC of 0.86 (95% CI: 0.74–0.98).

**TABLE 2:** Predictive performances of different machine learning classifiers.

Model	AUC	Accuracy	Sensitivity	Specificity
LASSO	0.82	0.79	0.77	0.81
LR	0.89	0.85	0.88	0.83
SVM	0.79	0.74	0.82	0.71
RF	0.69	0.59	0.55	0.62
DT	0.80	0.77	0.72	0.80
KNN	0.70	0.72	0.65	0.75
NB	0.65	0.57	0.60	0.55
AdaBoost	0.75	0.72	0.70	0.73

**FIGURE 4:**
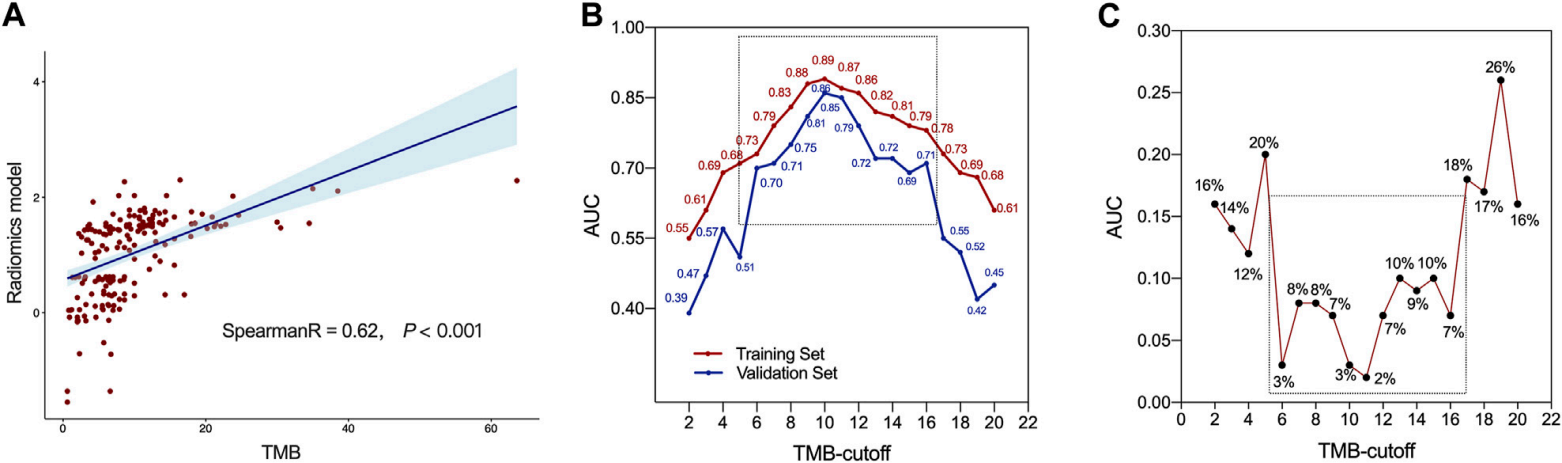
The predictive ability of radiomics model. **(A)** Correlation between radiomics model (RM) and tumor mutation burden (TMB) values. **(B)** The AUCs of RM at different threshold values of TMB. **(C)** Difference of AUCs between the training and validation sets with RM at different threshold values of TMB.

Spearman correlation analysis showed that RM and TMB level had a good correlation (R coefficient: 0.62, *p* < 0.001, Figure 4A). Furthermore, as a consensus on a universally accepted cutoff value for high-TMB in GC was lacking, we proceeded to examine the predictive efficacy of RM across a variety of TMB cutoff values, ranging from 1 to 20 mut/Mb. As show in Figure 4B, the RM showed favorable accuracy within the cutoff value range 6–16 mut/Mb in both sets with AUCs greater than 0.70. In addition, the difference of AUCs between two sets were less than 0.10, indicating the robust stability of the RM (Figure 4C).

### Explainability of radiomics model

**FIGURE 5:**
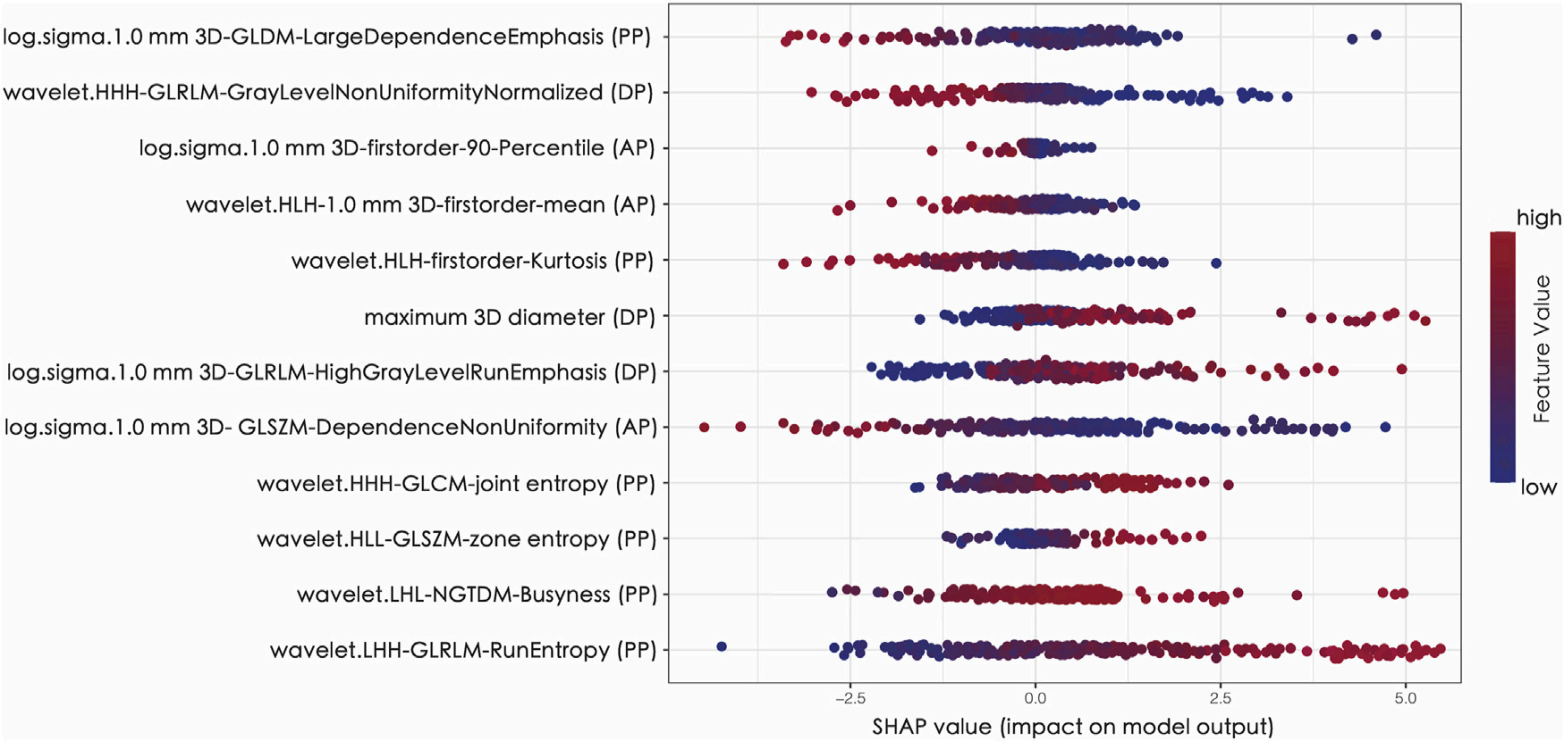
The Shapley additive explanations (SHAP) plot of features impact on predicted probability.

In order to assess the significance of features and enhance the comprehensibility of the RM, the SHAP values for the chosen features were calculated and displayed in the training dataset. Figure 5 shows that a positive SHAP value suggests a strong probability of detecting high TMB. (Figure 5).

## Discussion

In the current study, we proposed a ML-based CECT RM as a noninvasive image biomarker of TMB status in patients with GC. The RM exhibited precise discriminatory ability in both the training and validation sets.

Due to highly intratumoral heterogeneity, GC has distinctive characteristics in signal conducting metabolism, proliferation, invasiveness and treatment response, which allow for the increasing of phenotypic and functional differences in the progression of tumorigenesis. Therefore, intratumoral heterogeneity plays an important role in tumor progression, therapy resistance, and disease recurrence (Dagogo-Jack and Shaw, 2018). Through high-throughput algorithms, the radiomics method is capable of extracting invisible high dimensional features from medical images and thus allowed quantitative analysis, which facilitating the deciphering of distinct phenotypic differences within tumors (Aerts et al., 2014). Accumulating evidence has demonstrated the promising potential of radiomics methods for characterizing gene mutation status and tumor heterogeneity. Rossi et al. proposed a RM for detection epidermal growth factor receptor (EGFR) mutation for NSCLC patients (Rossi et al., 2021). Gao et al. reported that a radiomics signature based on CECT images could effectively predict expression status of human epidermal growth factor receptor 2 in GC (Ma et al., 2022). Tian et al. established a RM to predict TP53 status in laryngeal squamous cell carcinoma patients (Tian et al., 2022). Nevertheless, studies focus on the correlation between radiomics and TMB status are still rare. Wang et al. reported that radiomics features such as wavelet filtered first-order, GLRLM, GLCM and GLSZM features were correlated with the level of TMB in early-stage lung adenocarcinoma. However, their study employed a singular threshold value for high-TMB (4 mut/Mb), and only include 51 patients (Wang et al., 2019). To the best our knowledge, this is the first study that investigated the value of radiomics method for TMB prediction in GC.

Several studies have demonstrated that first order features were significantly associated with tumor mutation status of tumor (Sacconi et al., 2017; Song et al., 2020). According to a previous systematic review, it was also found that the first order statistics were the most reliable features for assessing tumor heterogeneity (Traverso et al., 2018). Three first order features (Kurtosis, mean and 90 Percentile) were selected in our RM. GLCM describes the second-order joint probability of two pixels with a certain spatial relationship in an image region. GLSZM quantifies gray level zones of image. Entropy is one of the most important features of GLCM and GLSZM. Joint entropy quantifies the level of the randomness or variability in neighborhood intensity values. Higher value of the joint entropy of the GLCM reflects the existence of diverse spatial relationships between pixels and therefore suggests a more heterogenicity within tumor. Zone entropy quantifies the level of uncertainty or randomness present in the distribution of zone sizes and gray levels. A higher value of zone entropy also serves as an indicator of increased heterogeneity within the texture patterns. Trebeschi et al. reported that GLSZM-Zone Entropy could be a predictor of immunotherapy efficacy of melanoma and NSCLC (Trebeschi et al., 2019). Lee et al. also found that entropy was the most crucial feature for prognostic prediction in breast cancer (Lee et al., 2022b). In line with previous studies, joint and zone entropy were identified as the key features for predicting TMB status in GC. In addition, due to the potential for larger tumors to possess a greater number of gene mutations, the maximum 3D diameter was selected in our RM. Wavelet transformed features have been suggested to be associated with gene mutation status by several studies. Liu et al. reported that 3D Wavelet decomposition could predict EGFR mutation in NSCLC. Song et al. reported Wavelet-LHH-GLDM-Large Dependence High Grey Level Emphasis was significant associated with ALK rearrangement in lung cancer (Song et al., 2020). Of note, six wavelet filtered features were included in our sRM, which decompose the original images in three different directions and may further reflect the spatial heterogeneity of tumor. Hence, our RM presents promising insights into the tumor microenvironment, demonstrating strong explanatory power for predicting TMB status.

Although TMB is a promising predictor for ICIs treatment response, highly varying distributions of TMB have been observed among different types of tumors. Consequently, the optimal TMB cutoff for each cancer type demonstrates significant variation (McNamara et al., 2020). McGrail et al. reported that one-size-fits-all high-TMB cut point could not applied for predicting ICIs efficacy for all types of tumors (McGrail et al., 2021). Several cutoff values have been used to define high TBM in GC by different studies (Wang et al., 2019; Duan et al., 2022). In 2020, pembrolizumab was approved for the treatment of metastatic GC with high-TMB (≥10 mut/Mb) by the US Food and Drug Administration. Wang et al. employed a threshold of 12 mut/Mb, which represented the upper 20th percentile of TMB, to delineate high-TMB cases (Wang et al., 2019). Therefore, additional research is necessary to establish the optimal threshold values of TMB for predicting the response to ICIs within distinct tumor types. In this case, we assessed the predictive capability of our RM across various TMB cut-off values. The results indicated that the RM exhibited a promising and stable predictive accuracy within the range of 6–16 mut/Mb.

Ono et al. reported that serum CEA level was associated with higher TMB in NSCLC (Ono et al., 2020). Kasi et al. found that the ratio of CA19-9/CEA could identify MSI in colorectal cancer (Kasi et al., 2020). However, in this study, we observed no correlation between TMB and the levels of four commonly used serum biomarkers in GC.

This study has some limitations. Firstly, similar to other retrospective studies, the potential for selection bias exists in the current analysis due to the limited subset of patients who underwent NGS test. Secondly, the assessment of radiomics features might lack consistency across scanners and institutions due to variations in the parameters employed by each. Thirdly, 3D lesion segmentation on three phases CECT images is a computationally complex and time-intensive task. Fourthly, this study was conducted in a single center. Therefore, our RM should be further validated by larger prospective multicenter studies.

In summary, our study showed the potential of three phases CECT-based radiomics approach to predict TMB status in GC. The RM exhibited good predictive efficiency and may provide an easy-to-use non-invasive image biomarker for prediction and surveillance of TBM in GC.

## Data Availability

The raw data supporting the conclusion of this article will be made available by the authors, without undue reservation.
