# Supplementary material for: A machine learning-based radiomics model for prediction of tumor mutation burden in gastric cancer
Source: Front Genet. 2023 Nov 6;14:1283090. doi: 10.3389/fgene.2023.1283090 (PMC10657897; doi:10.3389/fgene.2023.1283090)
Supplement: Supplementary file 1 [file DataSheet1.docx]

**Supplementary methods**

**1. CT image acquisition protocol**

To ensure the sufficient distension of stomach, 800–1000 mL water was administered orally prior to CT examination. The scan parameters were as follows: tube voltage,120 kVp; current: auto; field of view, 350 mm × 350 mm; matrix, 512 × 512; slice thickness, 1.25- or 1.5- mm. Arterial phase (AP) images were obtained following delays of 20-30 seconds after intravenous injection of contrast media (2.5 mL/s, 1.2 mL/kg; Omnipaque 300, GE Healthcare, Chicago, Illinois) via a syringe pump. After waiting for 60 and 120 seconds, the portal venous phase (VP) and delay phase (DP) were acquired.

**2. Radiomic feature extraction and pre-processing**

Before feature extraction, CT images were resampled into 1.0× 1.0 × 1.0 mm^3^ resolution using linear interpolation. To standardize the intensity range across scanners, Z-score normalization was utilized. Radiomic features were extracted from the original images of tumors and images transformed by wavelet and Laplacian of Gaussian (LoG) filtration. The features were categorized into first order statistics, shape, Gray Level Cooccurence Matrix (GLCM), Gray Level Run Length Matrix (GLRLM), Gray Level Size Zone Matrix (GLSZM), Neighbouring Gray Tone Difference Matrix (NGTDM) and Gray Level Dependence Matrix (GLDM). Original images were wavelet filtered with three directions, resulting eight different combinations of feature. LoG filtration images were generated with sigma = 1, 2, 3, 4 and 5 mm.

Features with more than 5% incomplete values (i.e., unexpected zeros and NA) were regarded as unstable and removed. Remainder of incompleteness were interpolated by their median feature values, outliers and extreme values were winsorized by nearest IQR respectively. Then, features were scaled into 0 to 1 by z-score transformation to ensure the comparability of the dynamic range of radiomic features before selection.

**3. Threshold values of serum biomarkers**

The test results of 4 serum biomarkers including cancer antigen (CA)19-9, CA242, CA72-4, and carcinoembryonic antigen (CEA) were obtained. The threshold values of CA19-9, CA242, CA72-4, and CEA were 37 U/mL, 20 U/mL, 6.9 U/mL, and 5.0 μg/mL, respectively [1].

**References**

[1] Z. Sun, N. Zhang, Clinical evaluation of CEA, CA19-9, CA72-4 and CA125 in gastric cancer patients with neoadjuvant chemotherapy, World J Surg Oncol 12 (2014) 397, https://doi.org/10.1186/1477-7819-12-397.
